# From trials to practice: Immune checkpoint inhibitor therapy for melanoma patients in Norway

**DOI:** 10.2340/1651-226X.2024.41266

**Published:** 2024-12-17

**Authors:** Denise Reis Costa, Anna K. Winge-Main, Anna Skog, Kaitlyn M Tsuruda, Trude Eid Robsahm, Bettina Kulle Andreassen

**Affiliations:** a Department of Research, The Cancer Registry of Norway, Norwegian Institute of Public Health, Oslo, Norway; b Norwegian Research Centre for Women’s Health, Oslo University Hospital, Oslo, Norway; c Department of Oncology, Oslo University Hospital, Oslo, Norway; d Oslo University, Faculty of Medicine, Oslo, Norway; e Registry Department, The Cancer Registry of Norway, Norwegian Institute of Public Health, Oslo, Norway

**Keywords:** Real-world data, melanoma patients, tumor characteristics, treatment management, clinical trials

## Abstract

**Background and purpose:**

Norway has one of the highest rates of cutaneous melanoma (CM) incidence and mortality globally. Immune checkpoint inhibitor (ICI) therapy for CM was introduced between 2014 and 2017 to improve treatment and patient prognosis, but knowledge about its clinical usage is limited. This study investigates patient’s characteristics and treatment patterns in real-world practice compared to clinical trial results.

**Material and methods:**

All adult (≥18) CM patients treated with ICI therapy in Norway from 2014 to 2021 were included, utilizing high-coverage data from multiple national registries to describe patients’ health, socioeconomic factors, and treatment management, stratified by first ICI therapy. We compared patient and tumour characteristics with findings from five randomized controlled trials (RCTs).

**Results:**

Among 2,083 patients receiving ICI therapy, 975 (47%) received nivolumab as their first treatment in the metastatic setting. Patients on combination therapy were younger and had higher education and income levels compared to those on monotherapy. Overall, real-world patients were older and had a higher incidence of brain metastases than those in RCTs. Approximately, 1 in 5 patients would have been excluded from RCTs due to pre-existing autoimmune diseases. Targeted therapy was the most common secondary systemic treatment after first-line PD-1 inhibitors.

**Interpretation:**

This study details ICI therapy in Norway, highlighting differences between real-world ICI users and clinical trial participants, raising questions about the effectiveness of this treatment for patients not eligible for trials.

## Introduction

Norway actively contributes in advancing cutaneous melanoma (CM) treatment through participation in multinational randomized controlled trials (RCTs), including CA184-169 [1], CheckMate 067 [2], and KEYNOTE-006 [3]. Given that the country continues to rank among the nations with the highest CM incidence and mortality rates in the world [4], it is crucial to ensure treatment availability and improve the patient prognosis associated with CM.

Early detection of the disease in a local stage (stage I and II) is vital. Surgery is the primary treatment for stages I–III CM [5] and can be curative for many. However, recurrence rates are substantial: up to 25% for stage II and 70% for stage III (local lymph node metastasis) posing fatal risks [6]. During the last decade, a dramatic improvement in both overall survival (OS) and progression free survival (PFS) has been shown for patients with stage III and IV (distant metastasis) CM through the implementation of immune checkpoint inhibitor (ICI) therapy (i.e. cytotoxic T-lymphocyte associated protein 4 [CTLA-4] antibody and programmed cell death protein 1 [PD-1] antibodies) and targeted therapy with BRAF and MEK inhibitors [7–9].

National reimbursement decisions by government-appointed body, *Nye metoder*’s Decision Forum [10], determine treatment availability in Norway. The CTLA-4 antibody ipilimumab [11] was the first checkpoint inhibitor approved for reimbursement in specialist health care in 2013, and implemented January 2014, followed by the PD-1 antibodies nivolumab and pembrolizumab in 2015, and combination therapy of ipilimumab and nivolumab in 2017. Adjuvant therapies with nivolumab and pembrolizumab [12, 13] were incorporated in 2019 for patients with local lymph node metastasis [14].

The prospective phase IV IPI4-study initiated ipilimumab use in Norway offering insights into real-world effectiveness and safety [15]. Unlike clinical trials, real-world studies have broader patient eligibility criteria, providing a better understanding of effects in real-world clinical practice. Despite the insights gained from the 151 patients included in the IPI4-study, there remains limited knowledge about the characteristics and treatment patterns of *all* patients with CM undergoing treatment with immunotherapy in Norway. This information is pivotal, not only for understanding the impact of new treatments on patient survival [16] but also for assessing potential disparities in the administration of ICI therapy across the diverse patient groups that present in general clinical practice [17, 18].

By using Danish data, Donia and colleagues [18, 19] concluded that patients with metastatic melanoma are underrepresented in RCTs. To further investigate this claim, the objectives of this population-based cohort study are threefold: (1) to provide a comprehensive overview of the demographic and clinical profiles of patients with CM in Norway across the different ICI therapies during 2014-2021, in both metastatic and adjuvant settings; (2) to compare patient outcomes in real-world settings with those in pivotal RCTs; and (3) to describe their treatment management, including additional therapies and second-line treatments. By linking data from multiple national registries, this study aims to deepen our understanding of these clinical profiles in practice and highlight how they differ from those in clinical trials.

## Material and method

### Data sources

We obtained data from the Cancer Registry of Norway (CRN) and Norwegian Melanoma Registry (NMR). The CRN holds complete and high-quality data about all cancer diagnoses nationwide, based on mandatory reporting from several independent sources [20]. The NMR was established in 2008 under the CRN, adding more histopathological data to each CM diagnosis [21]. From these registries, we obtained information on: sex, age, and region of residence (categorized as South-Eastern/ Western/Central/Northern). Information on tumour location and histological subtype was categorized as presented in Supplementary Table 1; ulceration (present/not present/unspecified); vessel infiltration (no/yes/unspecified); Breslow thickness in mm; summary stage at diagnosis (categorized as local disease (no metastasis), regional metastasis (regional lymph nodes, satellites and/or in transit metastasis), distant metastasis (non-regional lymph node and organ metastasis), and unspecified) [22]; TNM stage at diagnosis categorized according to AJCC 8th revision (stage I–IV); and second primary CM diagnosis (no/1/≥2). Brain metastases were identified through radiotherapy records or metastasis location data from the NMR. Statistics Norway provided data about household income 1 year prior to diagnosis (categorized as low income: <20th percentile/intermediate income: 20–80th percentile/high income: >80th percentile/unspecified) and highest obtained education (categorized as compulsory education (≤10 years)/intermediate education (11–13 years)/higher education (≥14 years)/unspecified). Metastasis stage was categorized as M0, M1a or M1b; M1c (any other distant organs); M1d (brain); Mx (not available) and retrieved from pathology and clinical notifications indicating whether metastasis or recurrence was found.

We retrieved immunotherapy use (ipilimumab, nivolumab, pembrolizumab, or combination therapy) and administration dates from CRN [23] and Norwegian Patient Registry (NPR). The NPR also provided comorbidity indices based on hospitalizations up to 4 years prior to diagnosis [24] and we categorized this information as No: No hospital admissions and 0/ Yes: ≥1. We gathered information on radiation therapy from the CRN database and utilized the NPR, the CRN systemic anti-cancer treatment databased and the Norwegian Prescription database (NorPD) to obtain information on chemotherapy. NorPD also provided information on targeted therapies. We categorized patients as having autoimmune disease(s) if they received any treatment registered to NPR or NorPD as categorized in in Supplementary Table 1 (by ICD-10 and ICPC-2) within 4 years of the first ICI therapy. All data were linked using each patient’s unique national identity number.

### Study population

Our cohort comprises all patients aged 18 or older registered in the CRN with a diagnosis of CM (ICD-10: C43), and who received at least one infusion of ipilimumab, nivolumab, pembrolizumab, or combination therapy (i.e. nivolumab and ipilimumab) from 2014 to 2021. Patients were followed for treatment paths until the end of the study (December 31, 2021), emigration, or death, whichever occurred first.

### Treatment regimens

The lines of therapy followed predefined algorithms based on Norwegian guidelines. First-line immunotherapy referred to the initial ICI treatment regimen after the primary CM diagnosis. In Norway, ipilimumab (3 mg/kg for both monotherapy and in combination with nivolumab), alone or with nivolumab (1 mg/kg in combination with ipilimumab), is administered for up to four doses. Monotherapy with pembrolizumab and nivolumab could extend treatment up to 2 years for metastatic patients and 1 year in the adjuvant setting. Adjuvant treatment was defined as those receiving treatment within 3 months after surgery of lymph nodes. A treatment line was advanced to the next line when a patient received new treatment including chemotherapy and targeted therapy.

### Clinical trials

We compared patient and tumour characteristics with findings from five relevant RCTs: CA184-169 (1), CheckMate066 (25), CheckMate067 (2), CheckMate238 (12), and KEYNOTE-006 (3).

### Statistical analyses

To describe patients, tumour characteristics, and treatment management, data were stratified by *first ICI therapy*. Additional information on tumour characteristics *at treatment initiation* (if available up to 6 months before treatment start) is presented in Supplementary Table 2. Descriptive statistics were provided as frequencies, percentages, medians, and interquartile ranges. All analyses were performed using R software (version 4.1.2).

## Results

### ICI treatment in Norway

**Figure 1:**
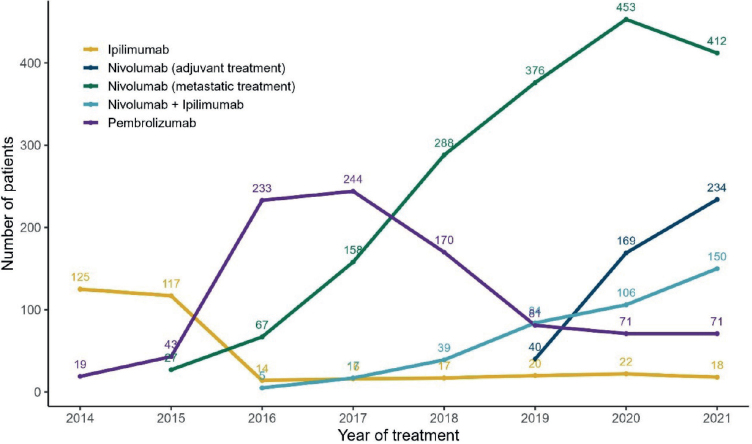
Number of patients receiving immune checkpoint inhibitor therapy for cutaneous melanoma in Norway, stratified by type and year of treatment. Patients undergoing continuous treatment are represented at multiple time points.

Figure 1 depicts the increasing use of nivolumab for metastatic treatment of CM in Norway over time. While most metastatic patients treated with ICI therapy between 2014 and 2015 were given the CTLA-4 antibody ipilimumab, preference shifted toward treatment with the PD-1 inhibitors nivolumab or pembrolizumab as monotherapy in subsequent years. Notably, by 2021, nivolumab monotherapy was the most used treatment, with the combination therapy of ipilimumab and nivolumab emerging as a secondary option for metastatic treatment. Treatment in the adjuvant setting was reimbursed in Norway from August 2019. Yearly price biddings determine the preferred drug of use in national hospitals and nivolumab was the PD-1 inhibitor that won the PD-1 inhibitor bids for the years 2019 through 2021. These two factors account for the sharp increase in the number of patients using nivolumab in recent years, with 40 patients receiving this treatment in 2019, rising to over 230 patients in 2021.

### Patient and tumor characteristics from patients treated in real-world clinical practice

Of the 2,083 CM patients treated with checkpoint immunotherapy during 2014-2021, more males than females received ICI therapy across all ICI treatments (Table 1). The median age for all patients treated with ICI was 68 years, with the oldest in the nivolumab metastatic treatment (median 71 years). Patients receiving combination therapy were more than a decade younger (median 58 years) than patients treated with ICI overall. ICI treatment was typically initiated 3 to 4 years after first CM diagnosis for metastatic patients and 2 years in the adjuvant setting.

**Table 1:** Patients’ characteristics who were treated with immune checkpoint inhibitor (ICI) therapy for cutaneous melanoma in Norway 2014–2021, stratified by their first ICI treatment.

Characteristics at treatment initiation	Total	Metastatic treatment				Adjuvant
		Ipilimumab	Pembrolizumab	Nivolumab	Nivolumab plus Ipilimumab	Nivolumab
No. (%)	2,083 (100)	202 (10)	383 (18)	975 (47)	203 (10)	320 (15)
Sex, No. (%)						
Female	770 (37)	74 (37)	136 (36)	348 (36)	85 (42)	127 (40)
Male	1,313 (63)	128 (63)	247 (65)	627 (64)	118 (58)	193 (60)
Age at treatment (years), median (IQR)	68 (58–75)	63 (53–69)	68 (59–75)	71 (62–78)	58 (49–66)	64 (54–74)
Time since initial diagnosis (years), median (IQR)	3 (2–6)	4 (3–6)	4 (2–6)	3 (2–6)	4 (2–7)	2 (2–2)
Region, No. (%)						
South-Eastern	1,317 (63)	118 (58)	244 (64)	624 (64)	126 (62)	205 (64)
Western	451 (22)	46 (23)	88 (23)	209 (21)	46 (23)	62 (19)
Central	254 (12)	24 (12)	40 (10)	118 (12)	21 (10)	51 (16)
Northern	61 (3)	14 (7)	11 (3)	24 (3)	10 (5)	2 (1)
Education level, No. (%)						
Compulsory education (≤ 10 years)	362 (17)	36 (18)	62 (16)	186 (19)	31 (15)	47 (15)
Intermediate education (11–13 years)	984 (47)	95 (47)	181 (47)	482 (49)	81 (40)	145 (45)
Higher education (≥ 14 years)	725 (35)	70 (35)	137 (36)	302 (31)	88 (43)	128 (40)
Unspecified	12 (1)	1 (1)	3 (1)	5 (1)	3 (2)	0 (0)
Household income, No. (%)						
Low	534 (26)	54 (27)	110 (29)	239 (25)	43 (21)	88 (28)
Intermediate	1,336 (64)	126 (62)	237 (62)	653 (67)	126 (62)	194 (61)
High	180 (9)	17 (8)	27 (7)	67 (7)	32 (16)	37 (12)
Unspecified	33 (2)	5 (3)	9 (2)	16 (2)	2 (1)	1 (0)
Commorbity, No. (%)						
No	1,840 (88)	188 (93)	346 (90)	831 (85)	186 (92)	289 (90)
Yes	243 (12)	14 (7)	37 (10)	144 (15)	17 (8)	31 (10)
Autoimmune diseases, No. (%)						
Yes	425 (20)	35 (17)	75 (20)	210 (22)	53 (26)	52 (16)
Brain metastasis, No. (%)						
Yes	299 (14)	59 (29)	72 (19)	134 (14)	24 (12)	10 (3)
TNM stage, No. (%)						
Progressed from I or II	574 (28)	62 (31)	104 (27)	298 (31)	55 (27)	55 (17)
III	484 (23)	25 (12)	58 (15)	173 (18)	26 (13)	202 (63)
IV	926 (45)	100 (50)	194 (51)	457 (47)	116 (57)	59 (18)
Unknown	99 (5)	15 (7)	27 (7)	47 (5)	6 (3)	4 (1)
First-line systemic treatment, No. (%)						
Immunotherapy	1,886 (91)	159 (79)	326 (85)	887 (91)	194 (96)	320 (100)
Targeted therapy	170 (8)	28 (14)	51 (13)	82 (8)	9 (4)	0 (0)
Chemotherapy	27 (1)	15 (7)	6 (2)	6 (1)	0 (0)	0 (0)

Most patients undergoing ICI therapy resided in the South-Eastern region (63%) and less than 4% were from Northern Norway. A higher percentage of patients with higher education started combination therapy (43%) compared to monotherapy in the metastatic setting (around 34%). High household income was more common among combination therapy patients (16%) and nivolumab adjuvant treatment (12%) than those on monotherapy metastatic treatment (around 8%).

Over 80% of CM patients undergoing ICI therapy had no comorbidities at diagnosis. Around 20% had an autoimmune disease, mainly among combination therapy patients (26%) and less frequent among ipilimumab monotherapy patients (17%) and adjuvant patients (16%). Nearly half (45%) were initially diagnosed with stage IV CM and 23% with stage III. Less than 29% had progressed to advanced stages from stages I-II. Brain metastasis occurred in 1 of 7 patients, higher among ipilimumab patients (29%) compared to other ICI treatments (3–19%).

Over 90% of the patients with CM initiated ICI treatment as their first systemic therapy. Targeted therapy was administered as a first systemic therapy to 8%, while fewer than 2% received chemotherapy. Among patients starting ICI treatment, approximately 15% received it in the adjuvant setting.

Characteristics of primary tumour at treatment initiation stratified by the type of first ICI treatment is shown in Supplementary Table 2. Most patients had a primary tumor located on the trunk (52%) and nodular was the most prominent histological subtype (52%). Approximately, 80% of patients who received combination therapy had distant metastasis and 13% had known brain metastasis.

### Patient and tumor characteristics in clinical trials

Table 2 shows differences in patient and tumor characteristics for CM patients treated with ICI therapy in real-world data versus those in pivotal RCTs. Generally, real-world patients were older and included a higher percentage of men in two out of eight RCT arms.

**Table 2:** Differences in key patient and tumor characteristics reported from selected clinical trials compared to our real-world data (RWD).

		Metastastic treatment							Adjuvant
		Ipi			Pembro	Nivo		Nivo + Ipi	Nivo
		CHECKMATE 067	KEYNOTE 006	CA184-169	KEYNOTE 006	CHECKMATE 067	CHECKMATE 066	CHECKMATE 067	CHECKMATE 238
Median Age (years)	RCT	62	62	62	63	60	64	61	56
	RWD	63	63	63	68	71	71	58	64
Males (%)	RCT	64	58	64	63	64	58	66	57
	RWD	63	63	63	65	64	64	58	60
Brain metastases (%)	RCT	5	10	17	10	3	3	4	0
	RWD	29	29	29	19	14	14	12	3
M0, M1a or M1b (%)	RCT	42	35	39	30	42	39	42	-
	RWD	32	32	32	40	39	39	39	-
Autoimmune disease (%)	RCT	0	0	0	0	0	0	0	0
	RWD	17	17	17	20	22	22	26	16
Stage III (%)	RCT	-	-	-	-	-	-	-	81
	RWD	-	-	-	-	-	-	-	78
Stage IV (%)	RCT	-	-	90	-	-	-	-	18
	RWD	-	-	88	-	-	-	-	21
Ulceration (%)	RCT	-	-	-	-	-	-	-	42
	RWD	-	-	-	-	-	-	-	46
Previous Chemo (%)	RCT	-	10	-	15	-	-	-	0
	RWD	-	8	-	2	-	-	-	0
Previous Targeted (%)	RCT	-	20	-	16	-	-	-	0
	RWD	-	14	-	13	-	-	-	0
Previous Radiation (%)	RCT	-	-	27	-	-	-	-	0
	RWD	-	-	23	-	-	-	-	1

Note: Highlighted cells show the disparities with an absolute difference ≥5 units, with darker shading for differences of ≥10 units. Blue-colored cells indicate lower values in our study compared to RCTs, while red-colored cells indicate higher values.

RCT: randomized controlled trial.

For metastatic treatment, a higher percentage of real-world patients had brain metastases compared to RCTs. For instance, 14% of nivolumab patients in our data had brain metastases versus 3% in CheckMate 067. Additionally, 40% of real-world patients were in metastasis categories M0, M1a, or M1b at treatment initiation with pembrolizumab compared to 30% in KEYNOTE-006. Real-world data also showed a high percentage of patients with autoimmune disease treated with ICI therapy, reflecting broader eligibility compared to RCTs.

In the adjuvant setting, the percentage of patients with an initial stage III diagnosis, ulceration presence and previous treatment was similar to CheckMate 238.

### Treatment management

The median time from initial diagnosis of advanced CM (i.e. stage III or IV) to initiating ICI treatment was 4 months (Table 3). Patients treated with nivolumab monotherapy in the adjuvant setting had the shortest median time to treatment initiation (3 months), while patients treated with pembrolizumab had the longest median lag time (9 months).

**Table 3:** Treatment management and additional treatment for patients with cutaneous melanoma in Norway 2014–2021, stratified by their first ICI treatment.

Characteristics	Total	Metastatic treatment				Adjuvant
		Ipilimumab	Pembrolizumab	Nivolumab	Nivolumab plus Ipilimumab	Nivolumab
No. (%)	2,083 (100)	202 (10)	383 (18)	975 (47)	203 (10)	320 (15)
Previous treatment – Radiation, No. (%)						
Yes, brain	77 (4)	9 (5)	28 (7)	38 (4)	2 (1)	0 (0)
Yes, no brain	210 (10)	38 (19)	57 (15)	95 (10)	16 (8)	4 (1)
Previous treatment – Chemotherapy, No. (%)						
Yes	29 (1)	16 (8)	7 (2)	6 (1)	0 (0)	0 (0)
Previous treatment – Targeted Therapy, No. (%)						
Yes	172 (8)	29 (14)	51 (13)	83 (9)	9 (4)	0 (0)
Most comphensive surgery, No. (%)						
Surgical removal of the tumor	1,700 (82)	170 (84)	310 (81)	793 (81)	147 (72)	280 (88)
Surgical removal of lymph nodes	91 (4)	6 (3)	16 (4)	26 (3)	8 (4)	35 (11)
Biopsy from metastasis, local recurrence or non-classified tumor	141 (7)	11 (5)	23 (6)	77 (8)	30 (15)	0 (0)
Therapeutic intervention directed at metastasis/palliative care	108 (5)	12 (6)	26 (7)	60 (6)	10 (5)	0 (0)
Other	43 (2)	3 (2)	8 (2)	19 (2)	8 (4)	5 (2)
Treatment management, First line, median (IQR)						
Treatment duration (months)	6 (2–12)	2 (1–2)	8 (2–22)	7 (2–12)	5 (1–14)	7 (3–11)
Time from advanced diagnosis to 1 L ICI treatment (months)	4 (3–12)	8 (4–17)	9 (2–16)	6 (2–18)	4 (1–16)	3 (3–4)
Time of last dose of 1L ICI treatment to death (months)	4 (2–11)	7 (3–19)	5 (2–11)	3 (1–9)	2 (1–5)	6 (3–8)

ICI: Immune checkpoint inhibitor.

Overall, the median duration of immunotherapy treatment for CM was 6 months, with ipilimumab lasting about 2 months and pembrolizumab 8 months. Across all first-line ICI therapy drugs, the median time from last first-line treatment dose to death from CM was approximately 4 months, with a lower median time for those combination therapy (2 months).

### Additional treatment

Less than 15% of the patients underwent radiotherapy before starting ICI therapy (Table 3). Notably, the percentage of patients receiving radiotherapy prior to ICI treatment was lower among those receiving adjuvant treatment (1%) or combination therapy (9%).

All patients who underwent ICI therapy also underwent at least one type of excision. Around 80% of the patients underwent ‘a surgical removal of the tumor’ as the most comprehensive surgery registered at the CRN.

### Second-line treatment for CM metastatic patients receiving first-line ICI therapy

**Figure 2:**
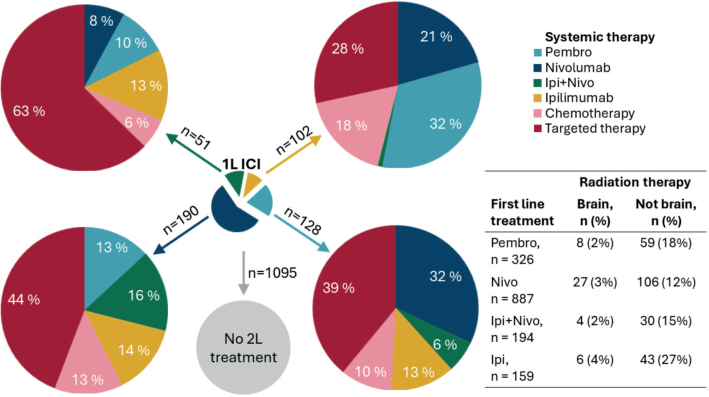
Second-line (2L) systemic (pie-charts) and radiotherapy (table) treatment for 1,566 cutaneous melanoma patients palliatively treated with first-line immune checkpoint inhibitor (ICI) therapy in Norway during 2014-2021. Pie-charts illustrate the proportion of 2L systemic treatment after 1L ICI: ipilimumab+nivolumab combination therapy (upper left), ipilimumab (upper right), nivolumab (lower left) and pembrolizumab (lower right). Patients who did not receive 2L systemic treatment until end of follow-up in December 2021 (n = 647) or death (n = 448) are illustrated in the 5th gray circle.

Of the 1,566 metastatic CM patients treated with first-line ICI therapy in Norway during 2014-2021, 471 (30%) received a second-line systemic treatment during the study period. Targeted therapy was the most common secondary systemic treatment after first-line pembrolizumab (39%), nivolumab (44%), and combination therapy (63%) (Figure 2, Supplementary Table 4). Pembrolizumab was the most frequent second-line choice following first-line ipilimumab (32%). For patients receiving radiotherapy after first-line ICI treatment, most of them received radiation to body parts other than the brain.

## Discussion

This study presents a retrospective panorama counting 2,083 patients receiving ICI therapy for CM patients in Norway from 2014 to 2021. By quantifying the number of patients receiving ICI therapies since their approval, along with their demographic and tumor characteristics and treatment paths, we highlight differences and similarities within the country, as well as with pivotal clinical trials [1–3, 12, 25] and international research [18, 19, 26, 27].

Consistent with patterns in CM incidence in the Norwegian population [20] and baseline characteristics in various Nordic real-world studies [18, 19, 26, 27], most patients receiving ICI therapy were male and over 60 years of age at the start of ICI therapy. Typical patients treated with combination therapy (nivolumab and ipilimumab) in Norway differed from those receiving monotherapy for metastatic treatment via ipilimumab, pembrolizumab or nivolumab in terms of median age (younger), sex (higher percentage of females), education level (higher education) and household income (higher income). This may be due to fact at the Norwegian guidelines (version 8) clearly stated that the improved PFS observed from the CheckMate 067 trial was only observed in patients <65 years old and with 59% having grade 3–4 adverse events, side effects were more frequent and more severe with combination therapy than monotherapy. Although our study could not assess it directly, higher income and education level may be associated with better physical condition, which may explain the characteristics of patients with CM being treated with combination therapy in the most recent years compared to other ICI therapies. We can also speculate if the higher educated group utilized centralized specialised treatment as combination therapy to a greater extent, as reported by Fiva et al. [28].

As anticipated, the patient profile in real-world clinical practice diverged from that in controlled RCTs, particularly among patients with brain metastases or pre-existing autoimmune diseases. CM has the highest risk of spreading to the brain among common cancer types [29], and clinical trials such as CheckMate 067, CA184-169 and KEYNOTE-006 excluded patients with symptomatic or active brain metastasis. The relatively strict criteria for inclusion of patients with brain metastases is reflected in the low number of these patients included in the RCTs compared to our data. Additionally, around 1 in 5 patients from our real-world population of CM patients would have been excluded from RCTs due to pre-existing autoimmune diseases.

Our study contributes to the existing literature by expanding the characterization of CM patients who received various ICI treatments. Notably, our findings complement those of the IPI4 study conducted in Norway [15], where baseline patient characteristics such as median age and sex distribution were replicated. However, the IPI4 study focused on the years 2014–2015, a period when ipilimumab was the sole approved ICI for CM in Norway.

In our study we identified a notable variation in first-line treatment choices among the analyzed ICI therapies. Notably, the percentage of patients receiving ipilimumab, including those from the IPI4 study, as first-line treatment was relatively low at 79% compared to other ICI therapies. Initially, limited availability of treatment options resulted in heavily pretreated patients. However, several other factors contributed to this trend. Firstly, ICI was available for all patients with inoperable or metastatic CM, unlike targeted therapy, which are applicable only to the 40–50% of patients that harbour the BRAF mutation [30]. Secondly, Norwegian guidelines specifically recommended PD-1 monotherapy for patients with inoperable or metastatic CM, both in the adjuvant setting and as the preferred first-line treatment. From its implementation in Norway in 2017 and throughout our study period the combination ICI therapy with ipilimumab and nivolumab was only reimbursed as a first-line treatment. In addition, the expanding indications for checkpoint inhibitors for other cancer types also raised awareness and acceptance of ICI therapy among both clinicians and patients. Lastly the use of ICI therapy observed in this study also reflects the healthcare framework in Norway, where full reimbursement is available for all CM patients undergoing this treatment. These findings underscore the evolving landscape of ICI therapy selection and highlight the impact of national guidelines and healthcare policies on treatment practices.

Despite little evidence supporting the benefit of switching between the two PD-1 inhibitors as they are perceived to be biosimilar [10], we found patients receiving nivolumab as second-line treatment after pembrolizumab and vice versa. The recent popularity of nivolumab for metastatic ICI may be partially explained due to its 4-week administration interval recommendation, compared to pembrolizumab’s 3 weeks, giving patients an extra week between treatments. The 2020 approval of a 6-week dosing regimen for pembrolizumab saw limited utilization in Norway. Furthermore, nivolumab has been the recommended drug since 2018 after national hospital-reimbursement biddings.

## Strengths and limitations

This study offers a comprehensive overview of ICI therapy utilization in Norway by incorporating extensive data coverage from the CRN, the NMR, the NPR and the NorPD. Utilizing these population-based registry data allows for a detailed analysis of patient demographic profiles and treatment specifics, facilitating a robust comparison with clinical trial data. In our analysis we also considered potential influencing factors from Statistics Norway, including education level and household income, to gain a deeper understanding of patients’ conditions. The study’s comprehensive data coverage on tumor characteristics and treatment management will also enable future investigations into survival trends within the Norwegian population.

A potential limitation of this study is the lack of detailed information regarding the processes underlying patient selection for ICI treatment. Geographic variations were observed, likely attributable to a relatively smaller population in the Northern region and possibly different disease profiles across the country [6]. A more detailed investigation is warranted to investigate these regional differences better.

There are some inherent limitations in the data sources used. NPR and NorPD are administrative databases that were not originally intended for research purposes. While NPR records reimbursement for services provided in public hospitals, NorPD contains data on dispensed drugs in Norway. Conversely, the CRN is specifically designed to collect information on administered anti-cancer treatments, but it currently lacks data from the Northern region. Incomplete registration of some variables might reduce the study population, however, given the high coverage of these population-based national registries, we believe that the missing data would not substantially affect our study’s findings. Furthermore, the duration of first-line treatment for patients initiating immunotherapy in the most recent period might be underestimated due to the study’s timeframe extending only until 2021. Despite these limitations we consider the study’s findings to be robust and informative due to the comprehensive and high-coverage nature of our data.

## Conclusion

This study provides a comprehensive overview of real-world patient profiles and treatment patterns for individuals with CM undergoing and ICI therapy in Norway. Notably, many patients treated in clinical practice would not have met the eligibility criteria for clinical trials, highlighting the need for further research to confirm the beneficial effects of RCT for this specific subgroup of patients.

In addition, it is essential to investigate how differences between patients treated with ICIs in clinical practice and those enrolled in RCT influence the overall benefit of these potent therapies. Consequently, our study provides valuable insights that can guide future research and contribute to the global effort to combat this challenging disease.

## Supplementary Material

From trials to practice: Immune checkpoint inhibitor therapy for melanoma patients in Norway

## Data Availability

Data sharing is not applicable due to the nature of the research and regulations.
