# Supplementary material for: From trials to practice: Immune checkpoint inhibitor therapy for melanoma patients in Norway
Source: Acta Oncol. 2024 Dec 17;63:41266. doi: 10.2340/1651-226X.2024.41266 (PMC11681278; doi:10.2340/1651-226X.2024.41266)
Supplement: From trials to practice: Immune checkpoint inhibitor therapy for melanoma patients in Norway [file AO-63-41266-s1.pdf]

Supplementary material has been published as submitted. It has not been copyedited, or typeset by Acta Oncologica

Supplementary Material

STable 1. International Classification of Diseases (ICD) codes for tumour location and histological subtype

Variable	ICD version	Codes
Tumour location	ICD-7	Head/neck (1400, 1401, 1408, 1409, 1442, 1900), Upper limbs (1902), Lower limbs (1903, 1904), Trunk (including perianal, 1901, 1905, 1907), Other (1908), Unspecified (1909)
Histological subtype	ICD-O-3	Lentigo maligna melanoma (87423), Nodular melanoma (87213), Superficially spreading melanoma (87433), Acral (87443), Other (87613, 87223, 87233, 87303, 87403, 87413, 87703, 87713, 87723), Unspecified (80003, 80703)

STable 2. Three-digit of the International Classification of Diseases (ICD) and International Classification of Primary Care, 2nd edition (ICPC-2) codes for autoimmune diseases

Autoimmune Diseases	ICD10	ICPC2
Rheumatologic		
Rheumatoid arthritis	M05, M06	L88
Psoriatic and enteropathic arthropathies	M07	
Connective tissue		
Systemic lupus erythematosus	M32	L99
Scleroderma (systemic sclerose)	M34	L99
Other (sjoegren syndrom, polymyalagia)	M35	L99
Dermatologic		
Psoriasis	L40	S91
Alopecia areata	L63	S23
Endocrine		
Graves thyroditis	E05	T85
Hashimoto thyroiditis	E06	T70
Diabetes mellitus type 1	E10	T89
Gastrointestinal		
Ulcerative colitis	K51	D93
Crohn's disease	K50	D93
Celiac disease	K90	D99
Neurologic		
Myasthenia gravis	G70	N99
Multiple sclerosis	G35	N86

STable 3. Tumour characteristics of patients at treatment initiation with primary cutaneous melanoma leading to immune checkpoint inhibitor (ICI) treatment in Norway 2014–2021, stratified by their first ICI treatment.

Characteristics at diagnosis	Total	Metastatic treatment				Adjuvant
		Ipilimumab	Pembrolizumab	Nivolumab	Nivolumab plus Ipilimumab	Nivolumab
**No. (%)**	637 (100)	26 (4)	69 (11)	232 (36)	56 (9)	254 (40)
**TNM stage, No. (%)**						
Progressed from I or II	32 (5)	1 (4)	8 (12)	20 (9)	1 (2)	2 (1)
III	282 (45)	2 (8)	14 (22)	60 (27)	9 (16)	197 (78)
IV	308 (50)	21 (88)	43 (66)	146 (65)	45 (82)	53 (21)
Unknown	15	2	4	6	1	2
Unspecified	6	0	0	4	1	1
**Tumor location, No. (%)**						
Head/neck	67 (16)	1 (7)	3 (8)	28 (23)	5 (24)	30 (14)
Upper limbs	39 (9)	1 (7)	3 (8)	10 (8)	0 (0)	25 (11)
Lower limbs	85 (21)	4 (29)	7 (19)	21 (18)	6 (29)	47 (21)
Trunk	214 (52)	7 (50)	22 (60)	60 (50)	10 (48)	115 (52)
Other	8 (2)	1 (7)	2 (5)	1 (1)	0 (0)	4 (2)
Unspecified	224	12	32	112	35	33
**Histological subtypes, No. (%)**						
Acral	10 (3)	1 (11)	0 (0)	2 (2)	0 (0)	7 (4)
Lentigo maligna melanoma	3 (1)	0 (0)	0 (0)	2 (2)	0 (0)	1 (1)
Nodular melanoma	163 (52)	5 (56)	13 (59)	48 (56)	6 (50)	91 (50)
Superficially spreading melanoma	129 (41)	2 (22)	8 (36)	29 (34)	6 (50)	84 (46)
Other	7 (2)	1 (11)	1 (5)	5 (6)	0 (0)	0 (0)
Unspecified	325	17	47	146	44	71
**Ulceration, No. (%)**						
Not present	167 (50)	4 (57)	11 (52)	36 (42)	6 (43)	110 (54)
Present	165 (50)	3 (43)	10 (48)	50 (58)	8 (57)	94 (46)
Unspecified	305	19	48	146	42	50
**Vessel infiltration, No. (%)**						
No	202 (82)	2 (100)	17 (94)	43 (69)	9 (75)	131 (86)
Yes	44 (18)	0 (0)	1 (6)	19 (31)	3 (25)	21 (14)
Unspecified	391	24	51	170	44	102
**Breslow thickness (mm), median (IQR)**	3 (2-6)	6 (4-14)	3 (1-4)	4 (2-8)	6 (2-10)	3 (2-5)
**Summary stage, No. (%)**						
No metastasis	23 (4)	1 (4)	7 (10)	13 (6)	1 (2)	1 (0)
Regional metastasis	301 (48)	4 (15)	19 (28)	68 (30)	10 (18)	200 (79)
Distant metastasis	308 (49)	21 (81)	43 (62)	146 (64)	45 (80)	53 (21)
Unspecified	5	0	0	5	0	0
**Second primary, No. (%)**						
No, only primary	549 (86)	23 (89)	56 (81)	202 (87)	53 (95)	215 (85)
Yes, one second primary	76 (12)	3 (12)	11 (16)	25 (11)	3 (5)	34 (13)
Yes, more than one second primary	12 (2)	0 (0)	2 (3)	5 (2)	0 (0)	5 (2)

Characteristics at diagnosis	Total	Metastatic treatment				Adjuvant
		Ipilimumab	Pembrolizumab	Nivolumab	Nivolumab plus Ipilimumab	Nivolumab
**Metastasis stage, No. (%)**						
M0, M1a or M1b	121 (37)	7 (32)	20 (40)	61 (38)	18 (39)	15 (28)
M1c (any other distant organs)	72 (22)	8 (36)	15 (30)	33 (21)	11 (24)	5 (9)
M1d (brain)	27 (8)	1 (5)	4 (8)	12 (8)	6 (13)	4 (7)
Mx (not available)	111 (34)	6 (27)	11 (22)	53 (33)	11 (24)	30 (56)

STable 4. Number of patients receiving or not receiving second-line systemic treatment after first-line ICI for metastatic treatment. Percentages are presented in parentheses.

1L ICI Treatment	Total	2L treatment						No 2L treatment	
		Chemotherapy	Ipilimumab	Nivolumab plus Ipilimumab	Nivolumab	Pembrolizumab	Targeted therapy	End of follow-up	Death
Ipilimumab	159 (100%)	18 (11%)	-	1 (1%)	21 (13%)	33 (21%)	29 (18%)	13 (8%)	44 (28%)
Nivolumab plus Ipilimumab	194 (100%)	3 (2%)	7 (4%)	-	4 (2%)	5 (3%)	32 (17%)	109 (56%)	34 (18%)
Nivolumab	887 (100%)	25 (3%)	26 (3%)	30 (3%)	-	25 (3%)	84 (10%)	436 (49%)	261 (29%)
Pembrolizumab	326 (100%)	13 (4%)	16 (5%)	8 (3%)	41 (13%)	-	50 (15%)	105 (32%)	93 (29%)
